# Synthesis and Preclinical Evaluation of [*Methylpiperazine*-^11^C]brigatinib as a PET Tracer Targeting Both Mutated Epidermal Growth Factor Receptor and Anaplastic Lymphoma Kinase

**DOI:** 10.1021/acs.jmedchem.3c00722

**Published:** 2023-08-30

**Authors:** Antonia A. Högnäsbacka, Alex J. Poot, Esther Kooijman, Robert C. Schuit, Maxime Schreurs, Mariska Verlaan, Wissam Beaino, Guus A. M. S. van Dongen, Danielle J. Vugts, Albert D. Windhorst

**Affiliations:** † Department of Radiology & Nuclear Medicine, Amsterdam UMC, Vrije Universiteit Amsterdam, De Boelelaan 1117, 1081HV Amsterdam, The Netherlands; ‡ Biomarkers & Imaging, Cancer Center Amsterdam, Amsterdam, The Netherlands

## Abstract

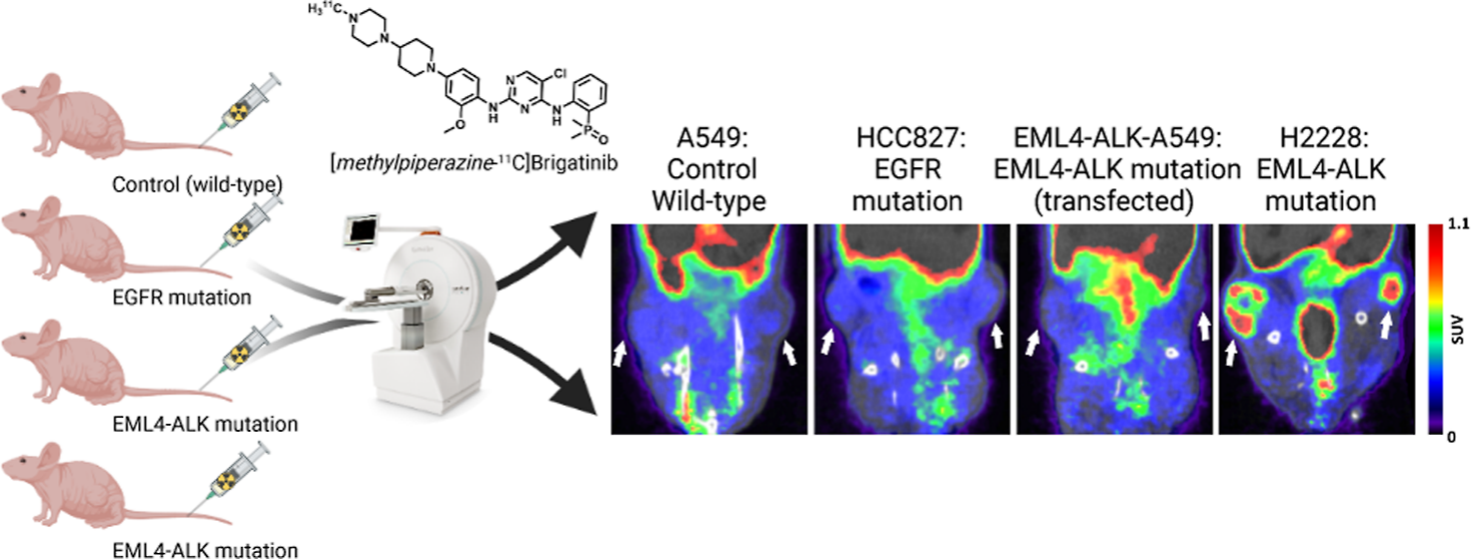

Brigatinib, a tyrosine kinase inhibitor (TKI) with specificity for gene rearranged anaplastic lymphoma kinase (ALK), such as the EML4–ALK, has shown a potential to inhibit mutated epidermal growth factor receptor (EGFR). In this study, *N*-desmethyl brigatinib was successfully synthesized as a precursor in five steps. Radiolabeling with [^11^C]methyl iodide produced [*methylpiperazine*-^11^C]brigatinib in a 10 ± 2% radiochemical yield, 91 ± 17 GBq/μmol molar activity, and ≥95% radiochemical purity in 49 ± 4 min. [*Methylpiperazine*-^11^C]brigatinib was evaluated in non-small cell lung cancer xenografted female nu/nu mice. An hour post-injection (p.i.), 87% of the total radioactivity in plasma originated from intact [*methylpiperazine*-^11^C]brigatinib. Significant differences in tumor uptake were observed between the endogenously EML4–ALK mutated H2228 and the control xenograft A549. The tumor-to-blood ratio in H2228 xenografts could be reduced by pretreatment with ALK inhibitor crizotinib. Tracer uptake in EGFR Del19 mutated HCC827 and EML4–ALK fusion A549 was not significantly different from uptake in A549 xenografts.

## Introduction

Lung cancer is one of the most common causes of cancer-related deaths, of which non-small cell lung cancer (NSCLC) accounts for approximately 85%.^1^ Part of the standard diagnostic evaluation of NSCLC patients is testing for receptor expression and mutational status in the lung tissue. Commonly found to be mutated in NSCLC adenocarcinomas are the epidermal growth factor receptor (EGFR) and the anaplastic lymphoma kinase (ALK).^2−4^ The most common EGFR mutations found are the tyrosine kinase domain mutations Del19 (deletion in exon 19) and L858R (insertion in exon 21),^5−7^ resulting in continuous receptor activation independent of ligand interaction. Patients with EGFR mutation-positive NSCLC are usually treated with tyrosine kinase inhibitors (TKIs) like gefitinib, erlotinib, or afatinib. However, treatment resistance in the form of the T790M mutation restores the constitutive activity of the receptor.^8−10^

NSCLC ALK mutations, on the other hand, are chromosomal rearrangements. The most common ALK mutation in NSCLC is caused by the echinoderm microtubule-associated protein-like 4 (EML4) gene’s 5′ end juxtaposing with the 3′ end of the ALK gene, resulting in the oncogene EML4–ALK. The resulting protein, thus, contains the amino-terminal half of EML4 and the intracellular catalytic domain of ALK.^11,12^ Patients with EML4–ALK mutation-positive NSCLC are treated with TKIs such as crizotinib. However, as observed with EGFR mutation therapy, treatment resistance in the form of point mutations also occurs in this type of NSCLC.^13^

Brigatinib is a tyrosine kinase inhibitor designed to inhibit a broad range of ALK kinase domain mutations, such as the F1174C/V, I1171N, and G120R mutations reported to occur as treatment resistance response to crizotinib or second-generation inhibitors like ceritinib and alectinib.^14,15^ However, brigatinib is also indicated for primary EML4–ALK mutation-positive NSCLC.^16^ Recently, brigatinib was proposed as a dual TKI as it also inhibited mutated EGFR *in vitro*.^17^ Thereby, multiple NSCLC mutation subtypes could benefit from brigatinib treatment. However, to predict which NSCLC patients benefit from brigatinib treatment, the mutational status of the tumors needs to be assessed. This is traditionally assessed through biopsy, which is invasive and sub-optimal due to tumor heterogeneity and recurring treatment resistance. PET-imaging with ^11^C-labeled brigatinib may provide a non-invasive method for assessing the mutational status, where the uptake of ^11^C-labeled brigatinib would imply a treatment-sensitive mutation.

Brigatinib is reported to be primarily metabolized by CYP2C8 and CYP3A4. Pharmacokinetic studies utilizing orally administrated [^14^C]brigatinib demonstrated the formation of two main metabolites, N-demethylated brigatinib and cysteine-conjugated brigatinib. *N*-Desmethyl brigatinib was considered the major metabolite at 3.5%, while unchanged brigatinib made up 92% of the circulating activity. Although the N-desmethylated metabolite is approximately 3-fold less potent than brigatinib,^16,18^ for PET, it is preferable to avoid target-binding radioactive metabolites as this could complicate image analysis and interpretation of the signal. Therefore, the methyl group expected to be removed upon metabolization was radiolabeled as this would lead to a non-labelled active metabolite, which is not an issue for image analysis.

Herein, we report the synthesis of [*methylpiperazine*-^11^C]brigatinib, and its preclinical evaluation as a PET tracer for imaging EML4–ALK and EGFR mutated NSCLC in female nu/nu mice carrying subcutaneous xenografts. Four xenografts were used. A549, which expresses wild-type EGFR/ALK, was used as a negative control [a cell growth inhibition concentration (IG_50_) of approximately 1 mM has been reported for brigatinib^17^]. Two EML4–ALK fusion gene-expressing xenografts were used; EML4–ALK fusion-A549 and H2228. EML4–ALK fusion-A549 is an isogenic cell line (ATCC CCL-185IG) transfected with the EML4–ALK fusion gene, which has been shown to have reduced cell survival compared to parental A549 when treated with ALK inhibitors crizotinib (IC_50_ of 663 nM) and ceritinib (IC_50_ of 6 mM).^19^ H2228, on the other hand, endogenously expresses the EML4–ALK fusion gene and is known to be brigatinib treatment sensitive^18^ with a cellular ALK phosphorylation IC_50_ of 4.5 ± 2.2 nM and a GI_50_ of 10.1 nM.^20^ Lastly, HCC827, which expresses del19 mutated EGFR, was used to evaluate uptake in EGFR mutated tumors, as it has been reported to be sensitive to brigatinib treatment, with a GI_50_ of 163 nM.^21^

## Results

### Precursor Synthesis

**Scheme 1:**
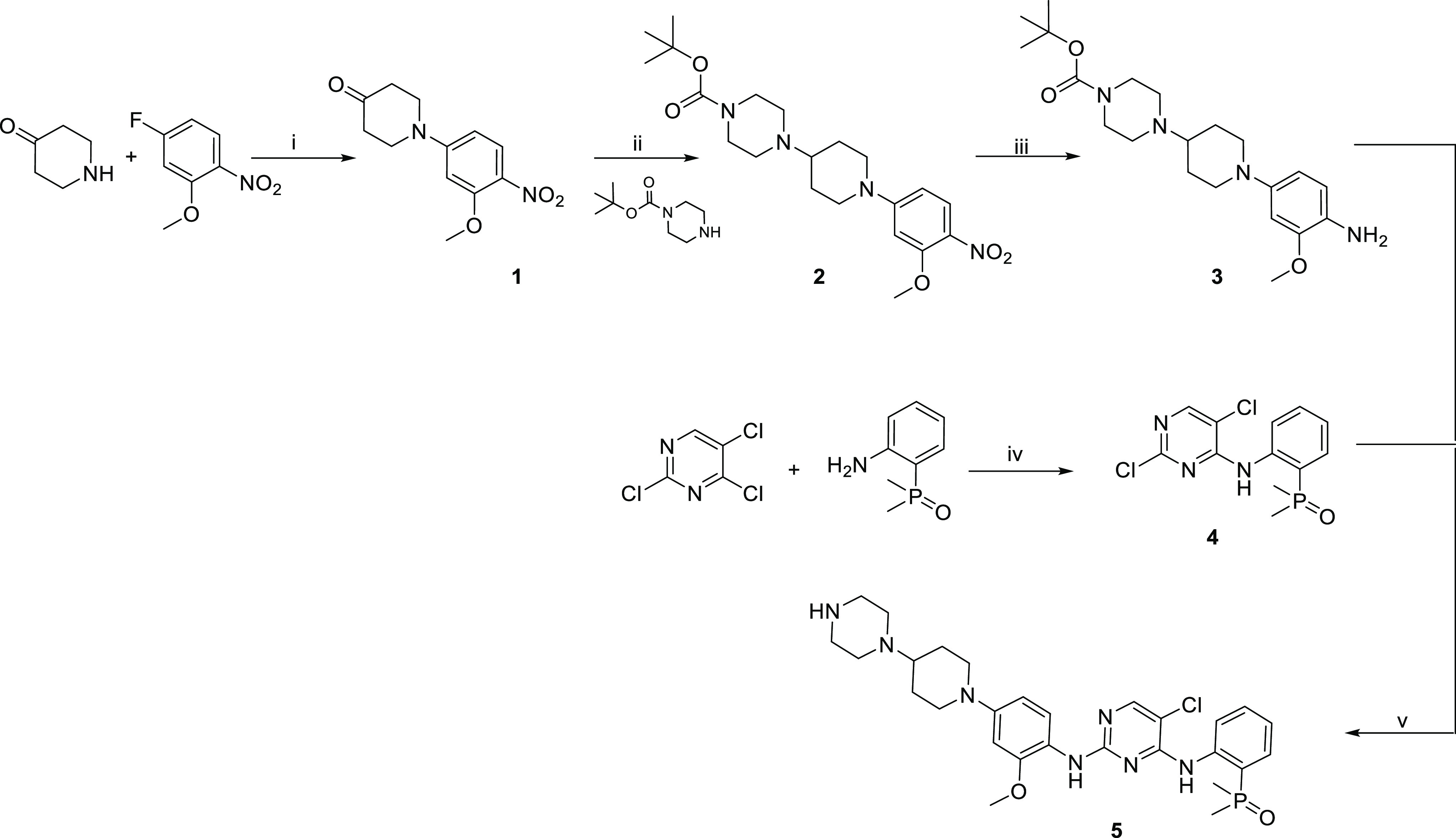
Synthesis of *N*-desmethyl brigatinib **5** Reagents and conditions: (i) potassium carbonate, dimethylformamide, 70 °C, overnight, 75%; (ii) triethylamine, acetic acid, sodium cyanoborohydride, toluene, room temperature, 2 + 3 h, 65%; (iii) 10% palladium on carbon, hydrogen gas (atm), ethanol, room temperature, 8 h, 94%; (iv) potassium carbonate, tetrabutylammonium hydrogensulfate, dimethylformamide, 65 °C, 8 h, 73%; (v) hydrochloric acid, 2-methoxy ethanol, 120 °C, 24 h, 23%.

*N*-Desmethyl brigatinib was synthesized according to literature procedures, as shown in Scheme 1.^15,22−24^*N*-Desmethyl brigatinib was obtained in an overall yield of 8% over five reaction steps.

### Radiochemistry

**Scheme 2:**
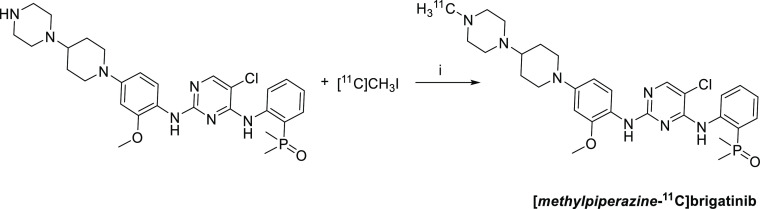
Radiosynthesis of [*methylpiperazine*-^11^C]brigatinib Conditions: (i) dimethyl sulfoxide/ethanol, 85 °C, 7 min, radiochemical yield 10 ± 2% (*N* = 15).

*N*-Desmethyl brigatinib was radiolabeled using [^11^C]methyl iodide without the addition of a base. By adding a protic solvent, the formation of an unknown byproduct could be suppressed while product formation was promoted. The side product-to-product ratio in dimethyl sulfoxide could almost completely be suppressed with the addition of ethanol, as is exemplified in the Supporting Information (Figure S1). The optimal conditions for the synthesis of [*methylpiperazine*-^11^C]brigatinib were thus found to be reacting *N*-desmethyl brigatinib with [^11^C]methyl iodide in a mixture of dimethyl sulfoxide and ethanol (1:1) at 85 °C for 7 min (Scheme 2). The product was purified by semi-preparative HPLC, followed by reformulation in saline with 10% ethanol. Extensive analysis of the pure HPLC product fraction in eluent revealed degradation of the product in acidic solutions over time. The formulated product, however, remained stable. An intravenous injectable solution of [*methylpiperazine*-^11^C]brigatinib was obtained in 49 ± 4 min with a radiochemical yield of 10 ± 2% (decay corrected to end of [^11^C]CO_2_ production), with a molar activity of 91 ± 17 GBq/μmol (at end of synthesis) and in a radiochemical purity of ≥95%.

### *In Vivo* Metabolism

**Figure 1:**
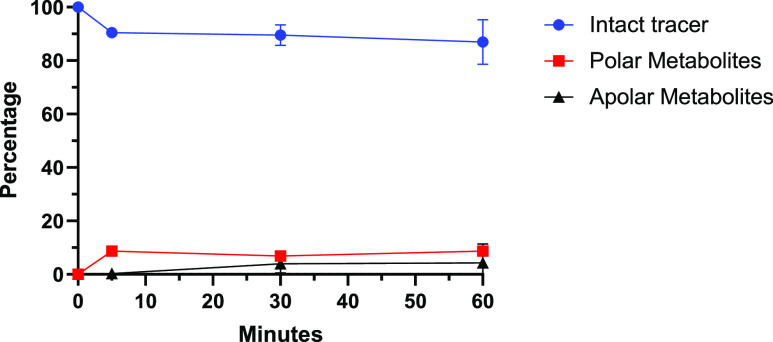
Metabolism of [*methylpiperazine*-^11^C]brigatinib in female nu/nu mice (*n* = 3 per time point).

The metabolism of [*methylpiperazine*-^11^C]brigatinib was evaluated in female nu/nu mice in conjunction with the biodistribution evaluation of the tracer in A549 tumor-bearing mice. At 60 min p.i., 87 ± 8% of the circulating radioactivity in blood plasma was intact tracer, while 9 ± 1% was polar metabolites and 4 ± 7% was apolar metabolites (Figure 1).

### *Ex Vivo* Biodistribution in A549 Xenografted Mice

**Figure 2:**
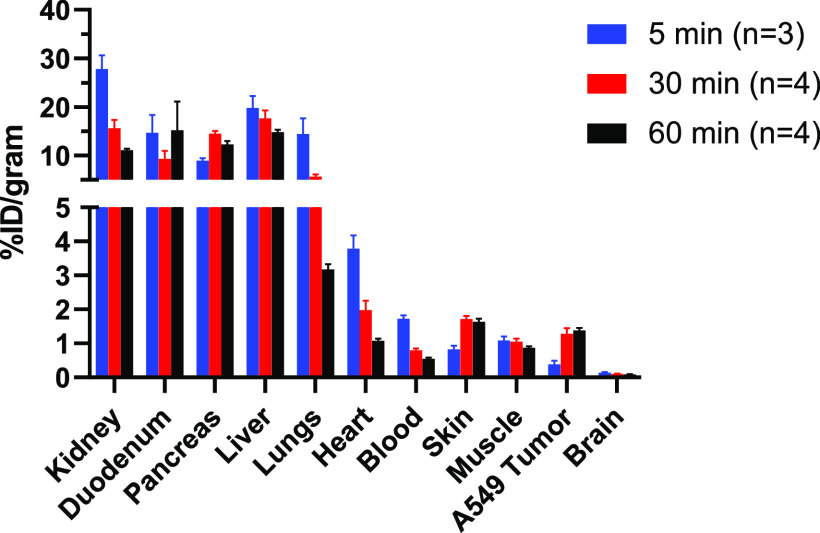
*Ex vivo* biodistribution of [*methylpiperazine*-^11^C]brigatinib at 5 (*n* = 3), 30 (*n* = 4), and 60 (*n* = 4) minutes p.i. in A549 tumor-bearing mice.

The biodistribution of [*methylpiperazine*-^11^C]brigatinib was evaluated at 5, 30, and 60 min p.i. in female nu/nu mice bearing wild-type EGFR and ALK expressing A549 xenografts. The tracer cleared rapidly from the blood (Figure 2), and high uptake of [*methylpiperazine*-^11^C]brigatinib could be observed in well-perfused organs like kidneys, liver, and lungs, as well as duodenum and pancreas shortly after tracer injection. However, the initial uptake observed in kidneys, lungs, heart, and blood decreased three- to five-fold at 60 min p.i. A very low radioactive concentration was observed in the brain. Both uptake and retention of radioactivity were observed for skin and muscle. The uptake and retention in A549 xenografts, which were chosen to represent ALK and EGFR mutation-negative tumors, were similar to that of skin.

### Tumor Uptake Comparison

**Figure 3:**
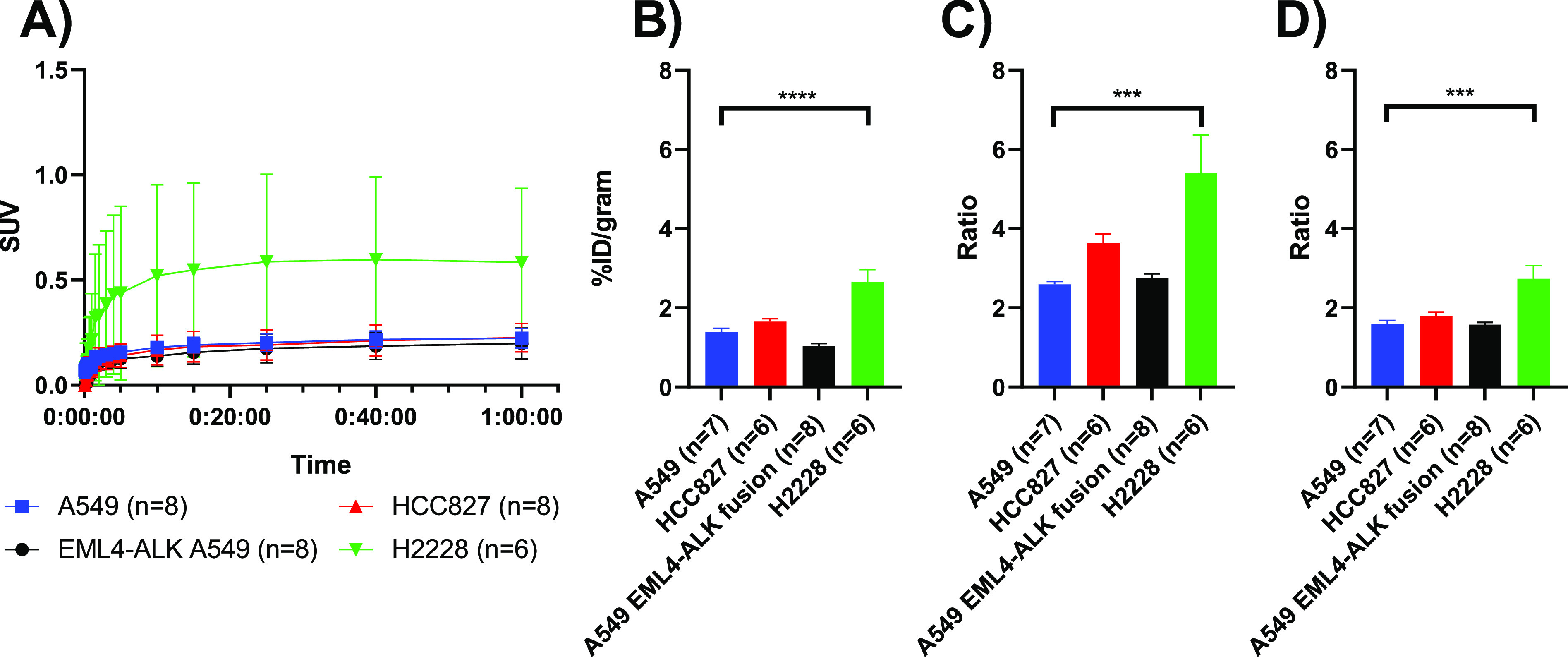
Tumor uptake of [*methylpiperazine*-^11^C]brigatinib in four xenograft models. (A) Time–activity curves assessed by PET, (B) tumor uptake at 60 min p.i., assessed by *ex vivo* biodistribution, (C) tumor-to-blood ratio at 60 min p.i., calculated from the *ex vivo* biodistribution, and (D) tumor-to-muscle ratio at 60 min p.i., calculated from the *ex vivo* biodistribution.

**Figure 4:**
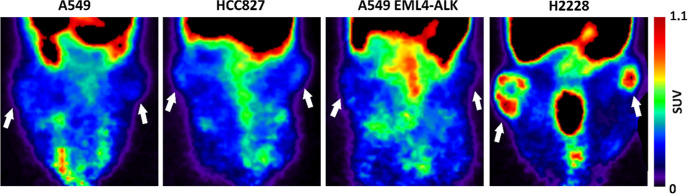
Representative PET images (static 0–60 min time frame) of [*methylpiperazine*-^11^C]brigatinib in NSCLC tumors (tumor locations indicated by white arrows).

The tumor-targeting potential of [*methylpiperazine*-^11^C]brigatinib was evaluated in four NSCLC cell lines; A549, HCC827, EML4–ALK fusion-A549, and H2228. Time activity curves are depicted in Figure 3A, demonstrating the H2228 xenograft to have the higher uptake. The *ex vivo* biodistribution at 60 min p.i. showed H2228 xenografts to have the highest uptake (2.65 ± 0.79 %ID/g), tumor-to-blood (T/B, 5.42 ± 2.32), and tumor-to-muscle (T/M, 2.74 ± 0.81) ratio (Figure 3B–D). This was significantly higher than the uptake in the control cell line A549 (1.40 ± 0.22% ID/g, 2.60 ± 0.20, 1.60 ± 0.22, respectively). Though the uptake in the EGFR Del19 mutated HCC827 (1.66 ± 0.17% ID/g, 3.64 ± 0.54, 1.79 ± 0.26, respectively) was higher compared to the control cell line A549, as observed in the *ex vivo* biodistribution, this was not statistically significant. The T/B and T/M ratios for A549 and A549 EML4–ALK fusion were very similar (A549: T/B 2.60 ± 0.20 and T/M 1.60 ± 0.22 *vs* EML4–ALK fusion A549 T/B 2.76 ± 0.29 and T/M 1.57 ± 0.18). Only the H2228 xenografts could be visually delineated with PET, and tracer uptake was observed to be heterogeneous within the tumor (Figure 4).

### Specificity of the Tumor Uptake of [*Methylpiperazine*-^11^C]brigatinib

**Figure 5:**
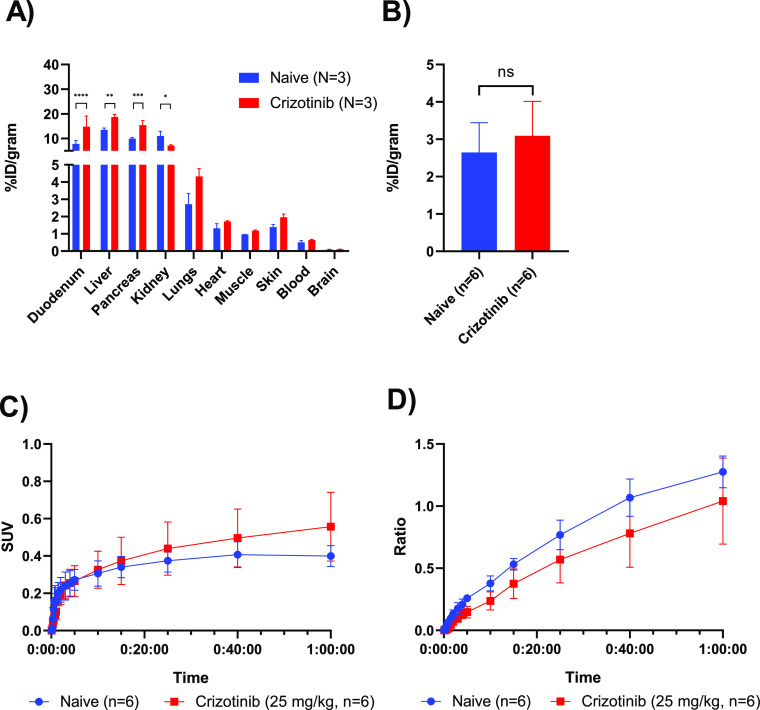
Biodistribution and tumor uptake of [*methylpiperazine*-^11^C]brigatinib in H2228 xenografts at baseline and following pretreatment with 25 mg/kg of crizotinib (*N* = 3 mice, *n* = 6 tumors). (A) *Ex vivo* biodistribution at 60 min p.i., (B) tumor uptake at 60 min p.i., assessed by *ex vivo* biodistribution, (C) time–activity-curve assessed by PET, and (D) tumor-to-blood ratio calculated from PET results.

To determine the specificity of the uptake of [*methylpiperazine*-^11^C]brigatinib in the H2228 xenografts, a PET/CT blocking study was performed using the EML4–ALK inhibitor crizotinib and the mutated EGFR inhibitor erlotinib (IC_50_ = 0.56 nM for wild-type EGFR^25^). After scanning, the mice were sacrificed, followed by an *ex vivo* biodistribution. The *ex vivo* biodistribution at 60 min p.i. revealed a significant increase in the radioactive concentration in the duodenum, liver, and pancreas following the pretreatment with crizotinib. A significant reduction, however, could be observed in the kidneys (Figure 5A). As the tumor uptake in the *ex vivo* and *in vivo* biodistribution was higher after pretreatment with crizotinib (Figure 5B,C), the tumor uptake was corrected to the blood activity concentration derived from the left ventricle of the heart. The tumor-to-blood ratio was significantly lower after pretreatment with crizotinib (*P*-value of 0.0083, Figure 5D).

**Figure 6:**
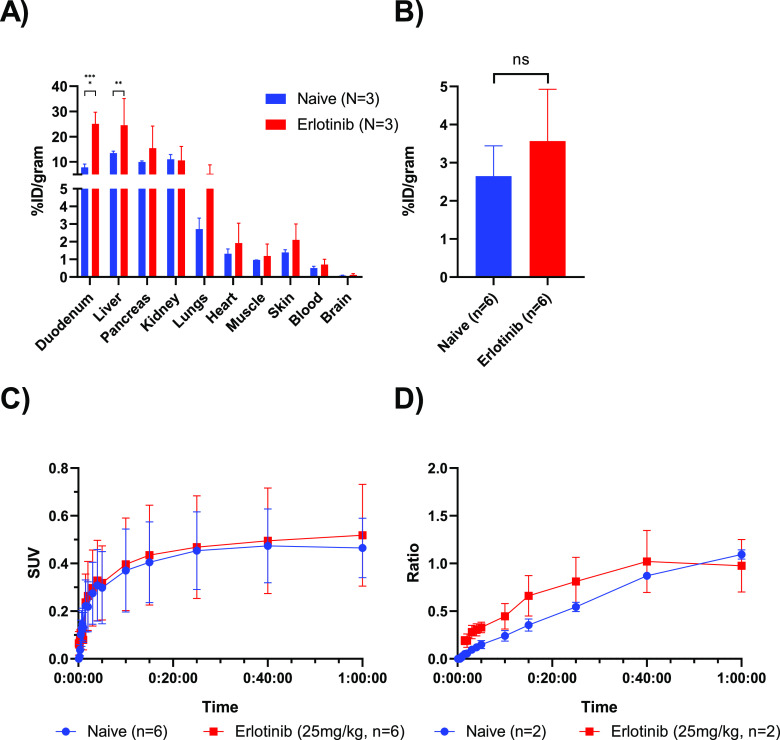
Biodistribution and tumor uptake of [*methylpiperazine*-^11^C]brigatinib in H2228 xenografts at baseline and following pretreatment with 25 mg/kg of erlotinib (*N* = 3 mice, *n* = 6 tumors, unless otherwise stated). (A) *Ex vivo* biodistribution at 60 min p.i., (B) tumor uptake at 60 min p.i., assessed by *ex vivo* biodistribution, (C) time–activity-curve assessed by PET, and (D) tumor-to-blood ratio calculated from PET results (*N* = 1, *n* = 2).

Similarly, an *ex vivo* biodistribution revealed a significant increase in the radioactive concentration in the duodenum and liver when mice were pretreated with erlotinib (Figure 6A). The tumor uptake did not change with erlotinib blocking, indicating the uptake to be less influenced by EGFR binding (Figure 6B,C). Due to experimental difficulties, the left ventricle could only be delineated for one mouse, which was needed to derive accurate blood activity concentration. Therefore, the effect of erlotinib on the tumor-to-blood ratio could not be determined (Figure 6D).

## Discussion

The present study was to determine the potential of brigatinib as a PET tracer for assessing the mutational status of NSCLC. The uptake of [*methylpiperazine*-^11^C]brigatinib was compared in the EML4–ALK mutated H2228 to that of the wild-type ALK and EGFR expressing A549 cell line in female nu/nu mice subcutaneous xenograft models. Tumor uptake, tumor-to-blood, and tumor-to-muscle ratios were significantly higher in the H2228 xenografts compared to the A549 xenografts. No difference was observed between the uptake in A549 and the EML4–ALK transfected A549 xenografts. A likely explanation is that the difference in EML4–ALK expression level is simply too small to be differentiated by PET or *ex vivo* distribution studies, as was indicated by the relatively high reported IC_50_ for crizotinib (IC_50_ of 663 nM) and ceritinib (6 mM) in EML4–ALK fusion-A549.^19^

Crizotinib, a first-generation ALK-inhibitor, was used to evaluate the specificity of [*methylpiperazine*-^11^C]brigatinib uptake in H2228 xenografts. Crizotinib is hypothesized to inhibit ALK in a similar spatial pocket as brigatinib and has been reported to inhibit ALK phosphorylation in H2228 cells with an IC_50_ of 55 ± 4 nM [albeit at a lower potency compared to brigatinib (4.5 ± 2.2 nM)].^20^ Initially, crizotinib pretreatment could not reduce the uptake of [*methylpiperazine*-^11^C]brigatinib in H2228 xenografts. This was hypothesized to be due to changes in the pharmacokinetics of the tracer under the EML4–ALK blocking conditions, as the tracer uptake was observed to differ in most organs, including blood. A significant decrease in tumor uptake was observed when the uptake was corrected against the radioactivity in the blood, indicating specific binding.

There was no significant difference in the uptake between the EGFR Del19 mutated HCC827 xenografts and the A549 xenografts, although the *ex vivo* results indicated a higher uptake in HCC827 xenografts. The uptake and retention of radioactivity in the skin and A549 xenografts were furthermore comparable. Most likely, the selectivity of brigatinib, between mutated and non-mutated EGFR, is not substantial enough to allow for *in vivo* differentiation. It could also be due to a lack of specific binding, low affinity, or the differences in expression levels in the xenografts could be too small to allow for *in vivo* differentiation. Our *in vivo* results could not confirm the previously reported *in vitro* findings.^17^

As for the biodistribution of [*methylpiperazine*-^11^C]brigatinib, very few metabolites were observed in plasma at 60 min. Of the metabolites circulating in plasma, the majority were polar, which would be consistent with the main metabolic pathway being demethylation, as expected based on the pharmacokinetic studies utilizing orally administrated [^14^C]brigatinib.^16,18^ The initial high uptake, followed by a rapid decrease in the kidneys would support renal clearance of the tracer.

High uptake of the tracer was observed in the duodenum. Part of this uptake could be EGFR binding, however, the uptake in the duodenum was 10-fold that of skin which has a high expression level of EGFR. Since also hardly any uptake in the brain was observed, brigatinib is most likely a P-glycoprotein (P-gp) substrate also at a tracer level,^18,26^ as P-gp is expressed both at the blood–brain barrier (transporting from the brain into the blood) and the duodenum (transporting from the blood toward the intestine).^27^

[*Methylpiperazine*-^11^C]brigatinib showed favorable characteristics in clearance from target organs. As the PET tracer would be primarily used to establish the mutational status of lung tumors, a low background would favor a high contrast. The observed high initial uptake in the lungs decreased to a fifth of the initial uptake at 60 min p.i., showing promise for the tracer to be used for lung imaging. The PET tracer could potentially be used to assess the mutational status and treatment sensitivity of metastases, of which the most common form is brain metastases.^28^ However, the brain uptake observed was very low, making it unlikely to be suitable for this purpose.

## Conclusions

The current study shows the successful synthesis of [*methylpiperazine*-^11^C]brigatinib, the preclinical evaluation in female nu/nu mice bearing subcutaneous NSCLC tumors, and the ability of [*methylpiperazine*-^11^C]brigatinib to differentiate between the EML4–ALK mutation expressing H2228 and the ALK wild-type expressing A549. The tumor-to-blood ratio in H2228 xenografts could be significantly reduced by crizotinib pretreatment, suggesting specific tracer uptake. A selectivity toward the EGFR Del19 mutated HCC827 over the wild-type EGFR expressing A549 was indicated but could not be proved. Nonetheless, the result obtained in this study is encouraging to further explore [*methylpiperazine*-^11^C]brigatinib as PET tracer targeting EML4–ALK mutations in human subjects.

## Experimental Section

### Precursor Synthesis

#### General

Chemicals and solvents were obtained from commercial sources; Merck/Sigma-Aldrich (Darmstadt, Germany), Fluorochem (Hadfield, United Kingdom), Fisher Scientific (Landsmeer, The Netherlands), Biosolve (Valkenswaard, The Netherlands), and Selleck Chemicals (Houston, TX, USA) and used without further purification. NMR spectra were recorded on a Bruker AVANCE II 500 Ascend (500 MHz for ^1^H, 126 MHz for ^13^C) or Bruker AVANCE III HD 600 (600 MHz for ^1^H, 150.92 MHz for ^13^C), at 20 °C. Chemical shifts (δ) are reported in parts per million (ppm) relative to the solvent [^1^H 2.50 ppm, ^13^C 39.52 ppm for dimethyl sulfoxide (DMSO)-*d*, ^1^H 7.26 ppm, ^13^C 77.16 ppm for chloroform (CDCl_3_)-*d*, and ^1^H 3.31 ppm for methanol (CD_3_OD)-*d*_4_]. Coupling constants (*J*) are reported in units of hertz (Hz). The following abbreviations are used to describe multiplicities: s (singlet), d (doublet), t (triplet), q (quartet), quin (quintet), m (multiplet), and br (broad). High-resolution mass spectra (HRMS, *m*/*z*) analyses were conducted on a Bruker microQTOF MS apparatus (capillary voltage: −4500 V; collision energy: 5 eV) using positive (ESI+) electrospray ionization. Thin-layer chromatography (TLC) was performed using TLC plates from Merck (aluminum TLC plates, silica gel coated with fluorescent indicator F254). Compounds on the TLC plate were visualized by UV light at 254 nm or by general TLC staining procedures if required. Flash column chromatography was performed on a Büchi Sepacore X10 flash system using silica packed cartridges. Aldrich silica gel 60A (230–400 mesh) was used for preparing pre-column cartridges.

##### 1-(3-Methoxy-4-nitrophenyl)piperidin-4-one (**1**)

To a mixture of 4-fluoro-2-methoxy-1-nitrobenzene (1.00 g, 5.84 mmol), potassium carbonate (2.02 g, 14.62 mmol) in dimethylformamide, piperidone monohydrate hydrochloride (0.99 g, 6.44 mmol) was added. The reaction mixture was stirred overnight at 70 °C. Water (30 mL) was added to the reaction mixture, and the resulting mixture was extracted with ethyl acetate (3 × 15 mL). The combined organic phases were washed with water and brine before drying over sodium sulfate. Solvents were removed, yielding an orange solid that was washed with diethyl ether and dried in a vacuum oven (1.10 g, 75% yield).

A mass spectra could not be obtained due to the compound not ionizing.

^1^H NMR (chloroform-*d*, 500 MHz): δ (ppm) 8.03 (d, *J* = 9.3 Hz, 1H), 6.45 (dd, *J* = 9.3, 2.6 Hz, 1H), 6.36 (d, *J* = 2.5 Hz, 1H), 3.97 (s, 3H), 3.80 (t, *J* = 6.2 Hz, 4H), 2.65 (t, *J* = 6.2 Hz, 4H).

^13^C NMR (chloroform-*d*, 126 MHz): δ (ppm) 206.47, 156.56, 153.94, 129.28, 105.10, 96.94, 56.48, 46.00, 40.12.

##### *tert*-Butyl-4-(1-(3-methoxy-4-nitrophenyl)piperidin-4-yl)piperazine-1-carboxylate (**2**)

1-(3-Methoxy-4-nitrophenyl)piperidin-4-one (1.00 g, 4.00 mmol) was suspended in 35 mL dry toluene. To it was added triethylamine (2.90 mL, 20.92 mmol), 1-boc-piperazine (1.49 g, 8.00 mmol), and acetic acid (1.80 mL, 31.47 mmol). The round bottom flask was equipped with an argon balloon, and the mixture was stirred at room temperature for 30 min. Sodium triacetoxyborohydride was added to the reaction mixture in batches with 30 min in between additions (2.03 g in total, 9.56 mmol). After the last batch, the reaction mixture was stirred for 3 h. A TLC confirmed the completion of the reaction. The reaction was carefully quenched with saturated sodium bicarbonate solution and stirred over the weekend. The resulting phases were separated, and the reaction mixture was extracted with a generous amount of dichloromethane. The combined organic phases were dried over sodium sulfate and concentrated *in vacuo*. The product was purified using flash column chromatography (0–5% methanol in dichloromethane). The product containing fractions were combined, and solvents were evaporated, yielding a hardened residue (1.09 g, 65%).

HRMS (ESI+, *m*/*z*): calculated for C_21_H_33_N_4_O_5_, 421.2445 (M + H); found, 421.2486.

^1^H NMR (DMSO-*d*_6_, 600 MHz): δ (ppm) 7.87 (d, *J* = 9.4 Hz, 1H), 6.58 (dd, *J* = 9.5, 2.6 Hz, 1H), 6.50 (d, *J* = 2.4 Hz, 1H), 4.02–4.08 (m, 2H), 3.90 (s, 3H), 3.25–3.30 (m, 4H), 2.90–2.97 (m, 2H), 2.51–2.55 (m, 1H, coincides with solvent), 2.43 (t, *J* = 4.9 Hz, 4H), 1.78–1.85 (m, 2H), 1.44 (qd, *J* = 12.3, 3.3 Hz, 2H), 1.39 (s, 9H).

^13^C NMR (DMSO-*d*_6_, 151 MHz): δ (ppm) 156.16, 155.10, 155.06, 153.73, 128.32, 127.52, 105.30, 96.70, 78.66, 60.56, 56.22, 48.49, 46.15, 44.48, 28.05, 27.24.

##### *tert*-Butyl-4-(1-(4-amino-3-methoxyphenyl)piperidin-4-yl)piperazine-1-carboxylate (**3**)

*tert*-Butyl 4-(1-(3-methoxy-4-nitrophenyl)piperidin-4-yl)piperazine-1-carboxylate (1.00 g, 2.38 mmol) was partly dissolved in 18 mL of dry ethanol. 10% palladium on carbon (0.25 g) was added to the flask, and the reaction mixture was vigorously stirred. Hydrogen gas at atmospheric pressure was introduced to the flask, and it was stirred at room temperature for 7.5 h. The reaction mixture was filtered through a pad of Celite. The solvent was evaporated *in vacuo*, yielding a lilac solid (0.87 g, 94%).

HRMS (ESI+, *m*/*z*): calculated for C_21_H_34_N_4_O_3_, 391.2704 (M + H); found, 391.2691.

^1^H NMR (chloroform-*d*, 600 MHz): δ (ppm) 6.63 (d, *J* = 8.3 Hz, 1H), 6.55–6.52 (m, 1H), 6.42 (d, *J* = 8.3 Hz, 1H), 3.83 (s, 3H), 3.76–3.34 (m, 5H), 2.87–2.39 (m, 6H), 2.12–1.51 (m, 6H), 1.46 (s, 9H).

^13^C NMR (chloroform-*d*, 151 MHz): δ (ppm) 148.10, 115.49, 110.17, 103.15, 55.64, 51.72, 48.93, 28.55.

##### (2-((2,5-Dichloropyrimidin-4-yl)amino)phenyl)dimethylphosphine Oxide (**4**)

In a round bottom flask tetrabutylammonium hydrogensulfate (56 mg, 0.16 mmol), (2-aminophenyl)dimethylphosphine oxide (277 mg, 1.64 mmol) and potassium carbonate (271 mg, 1.96 mmol) were suspended in dimethylformamide (4 mL). 2,4,5-Trichloropyrimidine (0.2 mL, 1.74 mmol) was added to the mixture, which was subsequently stirred at 65 °C for 8 h. A small amount of starting material could be detected, so the reaction mixture was stirred overnight at room temperature. The solvent was removed by evaporation, and the crude product was purified by flash column chromatography (0–5% methanol in dichloromethane). Fractions containing the product were combined, yielding a white solid (379 mg, 73%).

HRMS (ESI+, *m*/*z*): calculated for C_12_H_13_Cl_2_N_3_OP, 316.0168 (M + H); found, 316.0183.

^1^H NMR (DMSO-*d*_6_, 600 MHz): δ (ppm) 11.81 (s, 1H), 8.45 (s, 1H), 8.42 (dd, *J* = 8.5, 4.4 Hz, 1H), 7.59–7.69 (m, 2H), 7.25 (tdd, *J* = 7.6, 2.0, 1.0 Hz, 1H), 1.83 (s, 3H), 1.79 ppm (s, 3H).

^13^C NMR (DMSO-*d*_6_, 151 MHz): δ (ppm) 156.60, 156.58, 155.61, 142.04 (*J* = 2.6 Hz), 132.32 (*J* = 1.5 Hz), 127.40 (*J* = 1094.4, 10.5 Hz), 122.11, 121.66 (*J* = 7.2 Hz), 121.51, 114.87, 18.42, 17.95.

##### *N*-Desmethyl Brigatinib (**5**)

To a solution of *tert*-butyl 4-(1-(4-amino-3-methoxyphenyl)piperidin-4-yl)piperazine-1-carboxylate (50 mg, 0.13 mmol) in 0.5 mL, 2-methoxyethanol (2-((2,5-dichloropyrimidin-4-yl)amino)phenyl)-dimethylphosphine oxide (41 mg, 0.13 mmol) was added. The reaction mixture was stirred at room temperature for an hour before adding hydrochloric acid in isopropanol (6 N, 22 μL). The reaction mixture was heated to 120 °C for 23 h, at which point a TLC confirmed the reaction to have completed. The solvents were removed by evaporation, and the crude product was purified by column chromatography [8% ammonia in methanol (7 M) in dichloromethane]. Fractions containing the product were combined, and solvents were evaporated, yielding a brown solid (19 mg, 26%).

HRMS (ESI+, *m*/*z*): calculated for C_28_H_38_ClN_7_O_2_P, 570.2508 (M + H); found, 570.2523.

^1^H NMR (CD_3_OD, 500 MHz): δ (ppm) 8.34 (dd, *J* = 8.4, 4.5 Hz, 1H), 8.03 (s, 1H), 7.68–7.64 (m, 1H), 7.63–7.57 (m, 1H), 7.54–7.49 (m, 1H), 7.28–7.23 (m, 1H), 6.66 (d, *J* = 2.5 Hz, 1H), 6.45 (dd, *J* = 8.7, 2.6 Hz, 1H), 3.85 (s, 3H), 3.73–3.66 (m, 2H), 3.02 (t, *J* = 5.0 Hz, 3H), 2.80–2.66 (m, 6H), 2.48–2.36 (m, 1H), 2.03–1.97 (m, 2H), 1.84 (d, *J* = 13.5 Hz, 6H), 1.74–1.62 (m, 2H).^24^

### [*Methylpiperazine*-^11^C]brigatinib Synthesis

#### General Methods and Materials

Chemicals and solvents were obtained from commercial sources and used as received. Brigatinib, as a reference compound, was purchased from Selleck Chemicals (Houston, TX, USA). [^11^C]CO_2_ was produced by a ^14^N(p,α)^11^C nuclear reaction performed in a 0.5% O_2_/N_2_ gas mixture using an IBA Cyclone 18/9 cyclotron (IBA, Louvain-la-Neuve, Belgium). Subsequently, the [^11^C]CO_2_ was transferred to an in-house built synthesis unit and trapped in a reaction vial containing 0.1 mL of 0.1 M LiAlH_4_ in tetrahydrofuran. The reaction vial was heated to 130 °C under a helium flow to evaporate the tetrahydrofuran, after which 0.2 mL of 60% hydrogen iodide in water was added. The resulting [^11^C]CH_3_I was distilled and dried over a sodium hydroxide/Sicapent column before being introduced into a vial containing the reaction mixture.

Purification by semi-preparative isocratic high-performance liquid chromatography (HPLC) was performed using a Jasco PU-1587 station with a Jasco UV1575 UV detector (254 nm), a custom-made radioactivity detector, and chromatograms were acquired using Jasco ChromNAV CFR software (version 1.14.01, Tokyo, Japan).

The radiochemical purity and molar activity were determined by analytical HPLC, using a Shimadzu SPD20A system with either a Raytest 2 × 2 in. pinhole NaI-detector (Straubenhardt, Germany) or a Scionix Holland VD14-E1 (Bunnik, The Netherlands) detector and LabSolutions 5.85 software (Shimadzu Corporation, Japan).

Radioactivity amounts were measured using a Veenstra VDC-405 dose calibrator (Joure, The Netherlands). Radiochemistry was carried out in homemade, remotely controlled synthesis units. Radiochemical yields and molar activity were defined following the radiochemistry nomenclature guideline.^29^

#### [Methylpiperazine-^11^C]brigatinib

*N*-Desmethyl brigatinib (1.0 mg, 2.1 μmol) was weighed into a reaction vial and dissolved in 0.25 mL dimethylsulfoxide. [^11^C]CH_3_I was distilled into the reaction vial and 0.25 mL of ethanol was added to it, and the mixture was stirred vigorously. The reaction mixture was heated to 85 °C for 7 min, cooled to 30 °C, and diluted with 0.5 mL water. The product was purified by semi-preparative HPLC using a VisionHT C18 5 μm C18 250 × 10 mm column (Screening Devices BV, Amersfoort, The Netherlands) and 19:1:80:0.1 acetonitrile/tetrahydrofuran/water/trifluoroacetic acid as eluent (preconditioned with 19:1:80 acetonitrile/tetrahydrofuran/water). The collected product fraction was diluted with 60 mL water and passed over a tC18 Sep-Pak Light cartridge (conditioned with 10 mL ethanol and 10 mL water). After washing the cartridge with 20 mL of water, [*methylpiperazine*-^11^C]brigatinib was eluted with 0.6 mL sterile ethanol and diluted with 5.4 mL of saline.

The quality control of the injectable solution was executed on a Fortis UniverSil HS C18 5 μm 250 × 4.6 mm column with an eluent gradient of acetonitrile (A) in water (B), both containing 0.1% trifluoroacetic acid (0–2 min; 15%A, 2–10 min; 15–30%A, 10–13 min; 30%A, 13–18 min; 30–15%A) at a flow rate of 1 mL·min^–1^ at a UV wavelength of 254 nm (see Supporting Information Figure S2). The radiochemical purity of the product was ≥95%.

### *In Vivo* Evaluation

#### Cell Lines and Reagents

All cell lines were acquired from LGC (ATCC, Wesel, Germany). The human NSCLC cell lines A549 (CCL-185) and EML4–ALK fusion-A549 (CCL-185IG) were cultured in F-12K medium (Kaighn’s modification of Ham’s F-12 medium, Gibco, ThermoFisher Scientific, Waltham, MA, USA) supplemented with 10% fetal bovine serum (Gibco, Thermo Fisher Scientific, Waltham, MA, USA). HCC827 (CRL-2868) and H2228 (CRL-5935) were cultured in RPMI 1640 medium (ATCC modification, Gibco, ThermoFisher Scientific, Waltham, MA, USA) supplemented with 10% fetal bovine serum (Gibco, ThermoFisher Scientific, Waltham, MA, USA). All cell lines were maintained under standard cell culture conditions at 37 °C in a water-saturated atmosphere of 5% CO_2_ in air according to ATCC guidelines.

#### Xenografts

Female athymic nude mice (Hsd:Athymic Nude-Foxn1nu, 25 to 35 g, 7 to 8 weeks, Envigo, Horst, The Netherlands) were group-housed in pre-sterilized cages, provided with sterilized water and *ad libitum* access to Teklad mouse food, supplied with nesting material and kept under the standard animal room conditions (20–24 °C, 40–70% relative humidity, 12 h light/dark cycles). Animal experiments were performed in accordance with the European Community Council Directive (2010/63/EU) for laboratory animal care and the Dutch law on animal experimentation. The experimental protocol was validated and approved by the central committee for animal experimentation (CCD) and the local committee on animal experimentation of the VU University Medical Center. Animals were allowed to acclimatize for at least one week prior to the injection of tumor cells. Four cell lines [acquired from LGC (ATCC, Wesel, Germany)] were used in the study: A549, HCC827, EML4–ALK fusion A549, and H2228 (cell culture described in Supporting Information). The subcutaneous tumors were induced by injecting a suspension of 2.5 × 10^6^ cells in 100 μL phosphate-buffered saline in both flanks under isoflurane anesthetics (1–2% in oxygen). Once most tumors reached a suitable size (100 to 200 mm^3^), the mice were used for the studies. Due to the slow growth of the H2228 xenografts, the number of cells injected was increased to 4.5 × 10^6^ per flank for the blocking study.

#### *Ex Vivo* Biodistribution

Twelve A549 tumor-bearing female nu/nu mice were intravenously injected with 10 ± 3 MBq [*methylpiperazine*-^11^C]brigatinib under isoflurane anesthesia (2–2.5% in 1 L·min^–1^) and at 5, 30, 60 min p.i. sacrificed (*n* = 4 per time point). Lung, kidney, liver, duodenum, pancreas, heart, tail (site of injection), brain, blood muscle, skin, and tumors (left and right) were collected, weighed, and counted for radioactivity in a Wallac Compugamma 1210 counter (PerkinElmer, Turku, Finland). The percentage of the injected dose per gram of tissue (% ID/g) was calculated. Female nu/nu mice bearing EML4–ALK fusion A459, H2228, and HCC827 xenografts (*n* = 4 per xenograft) along with the A549 xenografted mice in the 60 min group were imaged by PET/CT prior to *ex vivo* biodistribution at 60 min p.i.

#### Metabolic Stability Study

Part of the blood (0.6–1 mL) collected *via* heart puncture in the biodistribution evaluation was used for the metabolic stability study. Centrifugation (4000 rpm for 5 min) separated the plasma from the blood cells. The sample was diluted with 0.15 M hydrochloric acid (2 mL) before loading onto an activated tC2 solid-phase extraction (SPE) cartridge. The filtrate and any remaining polar metabolites washed off the cartridge with water were collected and defined as the “polar” fraction. The apolar fraction was collected by eluting the cartridge with a mixture of methanol and water. An SPE extraction efficiency of 94 ± 2% was achieved. The collected fractions were counted for radioactivity in a Wizard gammacounter 2480 (Wallac/PerkinElmer, Waltham, MA, USA). The percentage of intact tracer was determined by semi-preparative radio-HPLC on a Dionex Ultimate 3000 system using a Phenomenex Gemini (250 × 10 mm, 5 μm) HPLC column and a gradient of 0 to 40% acetonitrile in water, both containing trifluoroacetic acid (0.1%), in 12 min at 3 mL·min^–1^. Fractions of 30 s were collected and counted for radioactivity in a Wizard gammacounter 1470 (Wallac/PerkinElmer, Waltham, MA, USA).

#### PET/CT in Xenografted Mice

PET/CT imaging was performed on a Mediso nanoScan PET/CT (Mediso Ltd., Hungary). Animals were kept under isoflurane anesthesia (1.5–2% in 1 L·min^–1^ oxygen) for the whole duration of the scans. Dynamic PET scans were immediately acquired for 60 min after tail vein injection of [*methylpiperazine*-^11^C]brigatinib. A computed tomography (CT) was performed just before the PET acquisition to acquire morphological data for image processing and reconstruction and identification of organs and tissue of interest. In the blocking study, the PET scan was followed by a contrast enhanced CT scan. The CT scan was acquired while infusing the contrast agent (Iomeron 400, Bracco Diagnostics, UK, 150 μL in 1 min) to visualize the cardiovascular structures.

PET scans were acquired in list mode and rebinned dynamically into the following frames: 4 × 5, 4 × 10, 2 × 30, 3 × 60, 2 × 300, 1 × 600, 1 × 900, and 1 × 1200 s, and statically in one frame (0–60 min), reconstruction was performed using a fully 3-dimensional reconstruction algorithm (Tera-Tomo, Mediso Ltd.) with 4 iterations and 6 subsets, and an isotropic 0.4 mm voxel dimension. PET image analysis and quantification were performed using VivoQuant software (version 2020, Invicro, Boston, MA, USA), and the data were expressed in SUV.

#### *In Vivo* Blocking

*In vivo* blocking was performed using the EML4–ALK blocking agent crizotinib and the mutated EGFR blocking agent erlotinib (25 mg/kg formulated in ≤200 μL, 5% v/v DMSO, 30% v/v PEG E 400, 65% v/v distilled water for injection and intravenously injected 1 h prior to the PET scan). H2228 tumor-bearing nu/nu mice (*n* = 3 per blocker) underwent two 60 min PET/CT scans, one baseline scan and one PET/CT scan an hour p.i. of the blocker. For each scan, the mice were intravenously injected with 11 ± 2 MBq of [methylpiperazine-^11^C]brigatinib. The mice were allowed to recuperate for a minimum of one day between the baseline and block scan.

### Statistical Analysis

Statistical analyses were carried out using GraphPad Prism version 8.0.2 software (GraphPad Software Inc., San Diego, CA). Results are expressed as mean values ± standard deviation. The tumor uptake, tumor-to-blood, and tumor-to-muscle ratios were analyzed for significance with a one-way ANOVA with Dunnett’s multiple comparison test, while significant differences between *ex vivo* tumor uptake between TKI pretreated (blocked) and naive H2228 xenografts (non-blocked) were analyzed with an unpaired *t*-test. The radioactive concentration in organs under these conditions was analyzed for their significant differences using a 2-way ANOVA with Sidak’s multiple comparison test.

The significance of the *in vivo* tumor-to-blood ratio difference between TKI pretreated (blocked) and naive H2228 xenografts (non-blocked) was analyzed by comparing the area under the curve for each tumor-to-blood ratio from 0 to 60 min in the dynamic PET using a one-tailed paired *t*-test.

## Supporting Information Available

Analytical HPLC comparison of crude reaction mixture when using ethanol as co-solvent; and an example of a typical HPLC chromatogram of [*methylpiperazine*-^11^C]brigatinib (PDF)
Molecular string of [*methylpiperazine*-^11^C]brigatinib (CSV)

## Abbreviations

ALK: anaplastic lymphoma kinase
EGFR: epidermal growth factor receptor
EML4: echinoderm microtubule-associated protein-like 4
IC_50_: half maximal inhibitory concentration
IG_50_: half-maximum growth inhibitory concentration
NSCLC: non-small cell lung cancer
p.i.: post-injection
TKI: tyrosine kinase inhibitor
T/B: tumor-to-blood ratio
T/M: tumor-to-muscle ratio
TLC: Thin-layer chromatography
SPE: solid-phase extraction
