# Supplementary material for: Synthesis and Preclinical Evaluation of [Methylpiperazine-11C]brigatinib as a PET Tracer Targeting Both Mutated Epidermal Growth Factor Receptor and Anaplastic Lymphoma Kinase
Source: J Med Chem. 2023 Aug 30;66(17):12130–40. doi: 10.1021/acs.jmedchem.3c00722 (PMC10510377; doi:10.1021/acs.jmedchem.3c00722)
Supplement: Supplementary file 1 — jm3c00722_si_001.pdf [file jm3c00722_si_001.pdf]

Supporting Information

Synthesis and preclinical evaluation of [*methylpiperazine*-^{11}C]brigatinib as a PET tracer targeting both mutated epidermal growth factor receptor and anaplastic lymphoma kinase

Antonia A. Högnäsbacka*[1,2], Alex J. Poot[1,2], Esther Kooijman[1,2], Robert C. Schuit[1,2], Maxime Schreurs[1,2], Mariska Verlaan[1,2], Wissam Beaino[1,2], Guus A.M.S. van Dongen[1,2], Danielle J. Vugts[1,2], Albert D. Windhorst[1,2]

[1]Amsterdam UMC, Vrije Universiteit Amsterdam, Dept. Radiology & Nuclear Medicine, De Boelelaan 1117, 1081HV, Amsterdam, The Netherlands
[2]Cancer Center Amsterdam, Biomarkers & Imaging, Amsterdam, the Netherlands

*Corresponding author: a.hognasbacka@amsterdamumc.nl

Contents

Analytical HPLC comparison of crude reaction mixture when using ethanol as co-solvent................ S2

An example of a typical HPLC chromatogram of [*methylpiperazine*-^{11}C]brigatinib............................. S3

Analytical HPLC comparison of crude reaction mixture when using ethanol as co-solvent

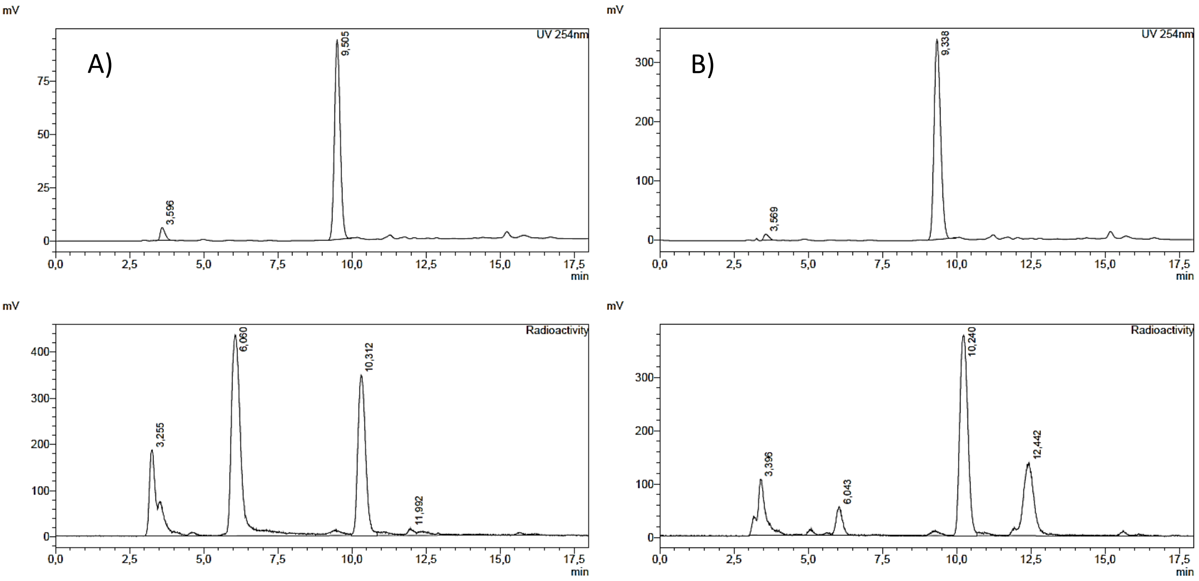

Figure S1: Analytical HPLC of the crude reaction mixture when A) the reaction was carried out in dimethyl sulfoxide (0.5 mL) versus B) the reaction was carried out in a mixture of dimethyl sulfoxide and ethanol (0.25 mL dimethyl sulfoxide and 0.25 mL ethanol), where [*methylpiperazine*-^{11}C]brigatinib has a radioactive retention time of 10.2-10.3 minutes.

An example of a typical HPLC chromatogram of [*methylpiperazine*-^{11}C]brigatinib

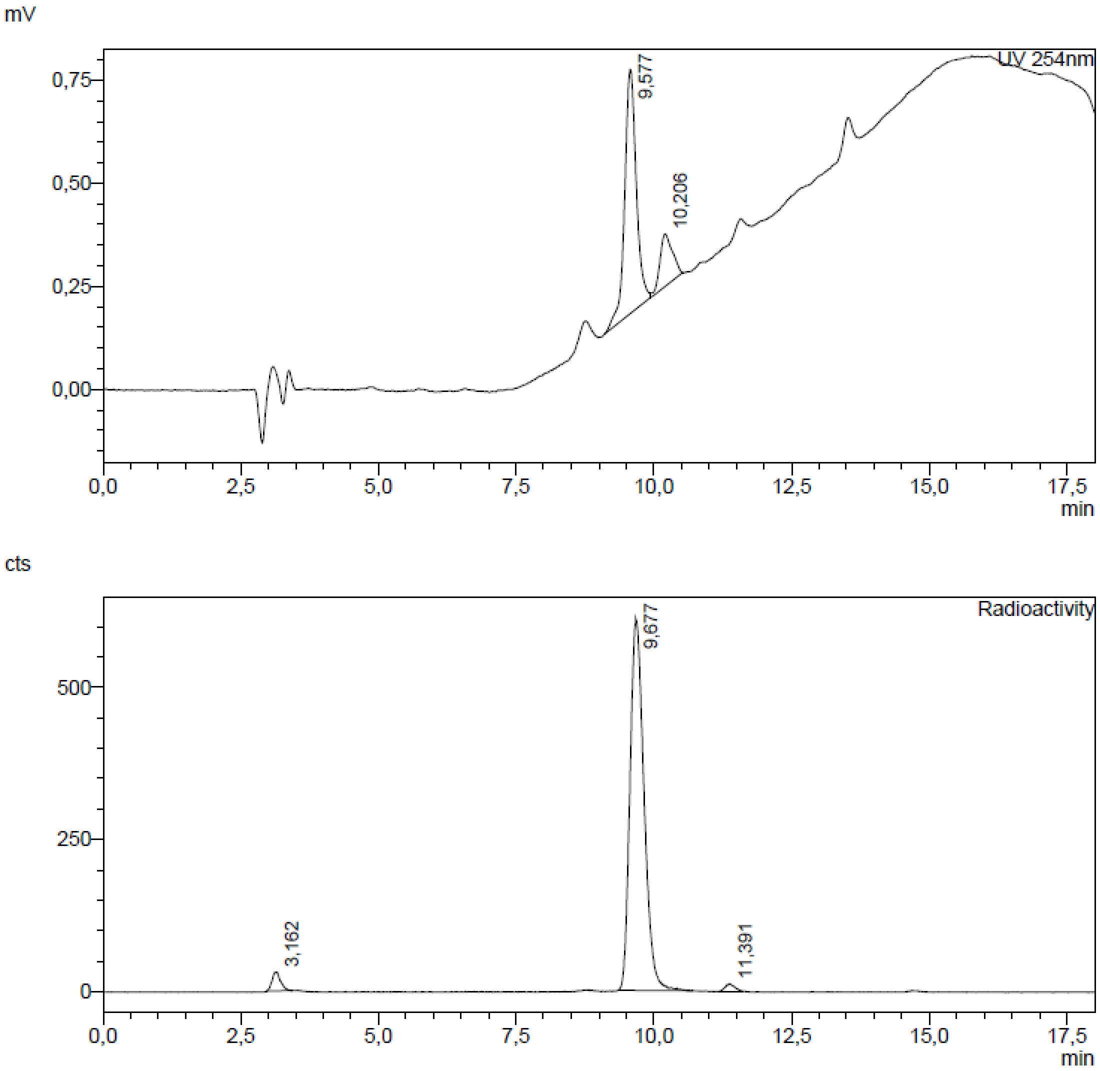

Figure S2. Radio-HPLC chromatogram of [^{11}C]brigatinib (UV retention time: 9.577, radioactive retention time: 9.677).
